# Clarity AD open-label extension data do not robustly confirm disease course modification by lecanemab in ApoE_4_ heterozygotes and non-carriers^☆^

**DOI:** 10.1016/j.tjpad.2026.100587

**Published:** 2026-05-06

**Authors:** Jemma Hazan, Kathy Y. Liu, Robert Howard

**Affiliations:** a Division of Psychiatry, University College London, London, UK; b North London NHS Foundation Trust, London, UK

**Keywords:** Alzheimer's disease, Dementia, Amyloid, Clinical trial

Perry and co-authors [1] present post-hoc subgroup analyses from the lecanemab Clarity AD core trial and the subsequent open-label extension in participants who were ApoE_4_ heterozygotes or non-carriers. This represented just over 80% of Clarity AD participants and at the 18-month primary outcome, lecanemab treatment was associated with an adjusted least-squares mean change difference from baseline of −0.58 (95% CI −0.81 to −0.35) CDR-SB points compared to placebo [2]. The authors conclude from the 18 to 36 months post-baseline open-label extension data that: “Delayed start results follow a parallel trajectory relative to early start results, but do not catch up, confirming a disease modifying effect and reflecting importance of early treatment initiation”.

This is the most ambitious claim from the data and will be important to prescribers, patients and their families because of the potential implications for “time savings” through delayed functional decline attributable to disease modification. However, the supporting open-label extension analyses were exploratory and descriptive, without pre-specified hypotheses or formal statistical testing. Although these outcomes were labelled as “delayed start results”, the study was not prospectively designed as a delayed start trial, which represents the most robust approach for testing a disease modification hypothesis. The mismatch between the strength of the authors’ conclusion and the exploratory nature of the underlying data is further underscored by the relegation of the open-label extension data to the supplemental materials (Figure S2).

Convincing demonstration of a disease modifying treatment effect with a prospectively designed delayed start study would require several elements that are not present in Clarity AD. Although the authors distinguish between “early start” and “delayed start” groups in Core and Extension phases, the 36-month outcomes must be interpreted in light of the fact that randomisation and treatment allocation blinding were not maintained during the open-label extension, rendering the analyses vulnerable to attrition and survivor bias, with no sensitivity analyses addressing these issues or how missing data was handled. In other words, their claim of disease modification rests on descriptive, post-hoc interpretation rather than pre-specified statistical criteria defining the degree of early treatment benefit that would need to be preserved to demonstrate disease-modifying effects [3]. Whilst open-label extension data can be beneficial for informing safety and tolerability over extended periods, findings should be reported responsibly, noting the limitations of the study design, to ensure accurate interpretation by the scientific, clinical, and patient community [4].

Despite these methodological limitations, we can see that at 36 months the adjusted least-squares mean change difference on the CDR-SB from baseline for continuous lecanemab treatment was 3.06 points, for delayed lecanemab treatment 3.55 points and for an external ADNI comparison group 3.92 points. The 36 months difference of −0.49 CDR-SB points between delayed and continuous lecanemab is smaller than at 18 months, but is it sufficiently robust to support the claim that delayed start participants do not catch up with those who received continuous treatment? To date, open-label extension data for all Clarity AD participants has only been presented graphically and without revealing absolute values for the CDR-SB or any measure of the variability of the distribution of data at 36 months and we are told only that standard error bars on the graph of decline in delayed and continuous treatment groups did not overlap [2]. This is surprising, as estimation of between-group differences was prespecified in the statistical analysis plan [2].

In the absence of data from a well-performed delayed start study, it would be difficult to claim disease modification with the level of robustness that would persuade a regulatory authority such as the FDA or EMA. However, if accompanied by appropriate acknowledgement of the limitations of design and interpretation, it would be important to understand if the CDR-SB differences between delayed and continuous treatment were statistically significant and at least *consistent with* delayed treatment participants not catching up with those who had started treatment earlier.

We would urge the authors and their Sponsor to publish the results of formal between-group testing (for the continuous and delayed lecanemab groups and not the ADNI one) or, at least, the 95% confidence intervals for the least-squares mean change difference from baseline to 36 months. If more detailed presentation of the data or further analyses can support an alternative interpretation we would encourage the authors and their Sponsor to either present this urgently or make anonymised individual-level data available for independent analysis.

## Funding

RH and KL are supported by National Institute for Health Research (NIHR) University College London Hospitals Biomedical Research Centre. JH is supported by the UCL ADAPT study (Blood Biomarker Challenge) and an Alzheimer’s Research UK Clinical Research Training Fellowship (CRTF2023B-003).

## Authorship / credit authorship contribution statement

All authors contributed to the conceptualization and writing of the manuscript.

## Declaration of the use of generative AI and AI-assisted technologies in scientific writing and in figures, images and artwork

No generative AI or AI-assisted technologies were used

## CRediT authorship contribution statement

**Jemma Hazan:** Conceptualization, Writing – original draft, Writing – review & editing. **Kathy Y. Liu:** Conceptualization, Writing – original draft, Writing – review & editing. **Robert Howard:** Conceptualization, Writing – original draft, Writing – review & editing.

## Declaration of competing interest

The authors declare the following financial interests/personal relationships which may be considered as potential competing interests: Jemma Hazan reports financial support was provided by 10.13039/501100002283Alzheimer’s Research UK. Kathy Y. Liu reports financial support was provided by NIHR University College London Hospitals Biomedical Research Centre. Robert Howard reports financial support was provided by NIHR University College London Hospitals Biomedical Research Centre. Robert Howard reports a relationship with Synaptogenix that includes: board membership. If there are other authors, they declare that they have no known competing financial interests or personal relationships that could have appeared to influence the work reported in this paper.
